# Supplementary material for: Causal Relationship between Aging and Anorexia Nervosa: A White-Matter-Microstructure-Mediated Mendelian Randomization Analysis
Source: Biomedicines. 2024 Aug 16;12(8):1874. doi: 10.3390/biomedicines12081874 (PMC11351342; doi:10.3390/biomedicines12081874)
Supplement: Supplementary file 1 [file biomedicines-12-01874-s001.zip › Supplementary Figure/Figure S4/Singe SNP plot of MR analysis for TL to ubm-b-1502.pdf]

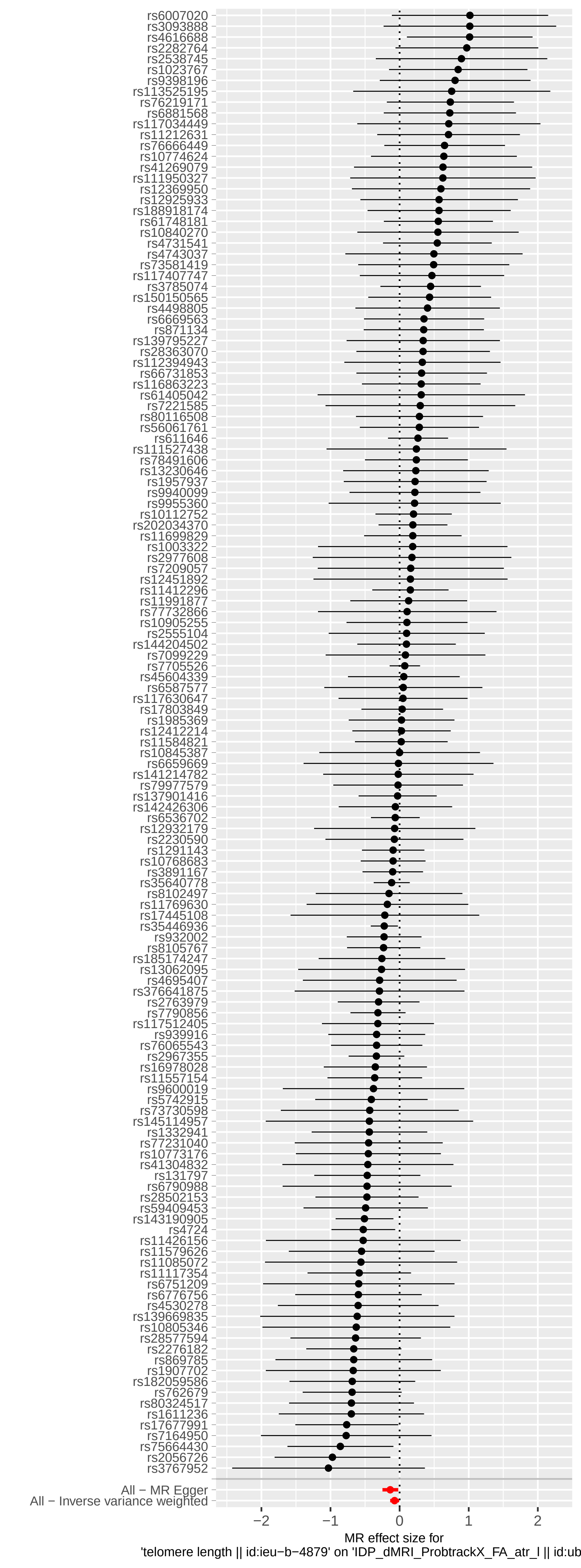

rs6007020
rs3093888
rs4616688
rs2282764
rs2538745
rs1023767
rs9398196
rs113525195
rs76219171
rs6881568
rs117034449
rs11212631
rs76666449
rs10774624
rs41269079
rs111950327
rs12369950
rs12925933
rs188918174
rs61748181
rs10840270
rs4731541
rs4743037
rs73581419
rs117407747
rs3785074
rs150150565
rs4498805
rs6669563
rs871134
rs139795227
rs28363070
rs112394943
rs66731853
rs116863223
rs61405042
rs7221585
rs80116508
rs56061761
rs611646
rs111527438
rs78491606
rs13230646
rs1957937
rs9940099
rs9955360
rs10112752
rs202034370
rs11699829
rs1003322
rs2977608
rs7209057
rs12451892
rs11412296
rs11991877
rs77732866
rs10905255
rs2555104
rs144204502
rs7099229
rs7705526
rs45604339
rs6587577
rs117630647
rs17803849
rs1985369
rs12412214
rs11584821
rs10845387
rs6659669
rs141214782
rs79977579
rs137901416
rs142426306
rs6536702
rs12932179
rs2230590
rs1291143
rs10768683
rs3891167
rs35640778
rs8102497
rs11769630
rs17445108
rs35446936
rs932002
rs8105767
rs185174247
rs13062095
rs4695407
rs376641875
rs2763979
rs7790856
rs117512405
rs939916
rs76065543
rs2967355
rs16978028
rs11557154
rs9600019
rs5742915
rs73730598
rs145114957
rs1332941
rs77231040
rs10773176
rs41304832
rs131797
rs6790988
rs28502153
rs59409453
rs143190905
rs4724
rs11426156
rs11579626
rs11085072
rs11117354
rs6751209
rs6776756
rs4530278
rs139669835
rs10805346
rs28577594
rs2276182
rs869785
rs1907702
rs182059586
rs762679
rs80324517
rs1611236
rs17677991
rs7164950
rs75664430
rs2056726
rs3767952
All − MR Egger
All − Inverse variance weighted
−2
−1
0
1
2
MR effect size for
'telomere length || id:ieu−b−4879' on 'IDP_dMRI_ProbtrackX_FA_atr_l || id:ub
